# Beyond the computational-representational brain: why affective neuroscience tells us attitudes must be explained on multiple levels

**DOI:** 10.3389/fnbeh.2014.00419

**Published:** 2014-12-04

**Authors:** David Haslacher

**Affiliations:** Artificial Intelligence, Department of Psychology and Department of Information and Computing Sciences, Utrecht UniversityNetherlands

**Keywords:** affective neuroscience, emotion, somatic markers, computational theory of mind, explanatory pluralism, attitudes, topographic maps, complex systems

A popular conception in the mind/brain sciences today is the metaphor of the brain as a computer. According to the computational theory of mind, all relevant cognition consists of executing some well-defined rules over representations which model (and have a direct correspondence to) the external world (Chalmers, 1996). I would like to challenge this notion and advocate a pluralistic/biological approach to cognition, specifically with respect to explaining emotions. In the following, I take explanatory pluralism to be the program of explaining complex phenomena by theories operating on multiple levels of measurement/explanation. I furthermore suggest that theories should be systematically connected (reduced by showing implementation, if possible) to theories at other levels. The necessity of biological modeling is probably self-evident to most neuroscientists. However, many theories in cognitive science and psychology present a certain functional constellation of which it is not clear how/if it relates to lower levels. These theories fall under the algorithm-oriented framework of cognitivism, which guides the study of thought in terms of abstract processes in some architecture of the mind/brain.

A framework which emphasizes the body in place of abstract processes—embodied cognition—has gained traction in the last decades and seems to have an indispensable advantage over cognitivism in that it allows for explanations which span multiple levels. Furthermore, it allows these explanations to be embedded in the body and environment. I argue that affective neuroscience has revealed that emotions are such an embodied phenomenon requiring description on multiple levels—including (but not limited to) the neurochemical, the systemic, and behavioral. I conclude that the use of algorithmic explanation (as opposed to simulation) should be limited and subject to verification within such a pluralistic epistemological landscape. In the following, drives are intended to denote homeostatic behavioral processes which are dependent on interoceptive representation. Emotions are intended to denote an affective state with cognitive, behavioral, and embodied components, thus subsuming drives. Attitudes are intended to denote long-term behavioral (especially object-directed) tendencies resulting from a history of emotional experiences.

## 1. Emotion: a multifaceted phenomenon

Modern embodied theories of emotions do not conceive of the process in a linear top-down or bottom-up fashion. Rather, causation is circular, such that cognition can trigger embodied reactions as well as vice versa. Thus, affective processes are nonlinear and situation-dependent. Furthermore, affective processes are now seen to involve a distributed set of brain systems with many causal pathways (Levine, 2009). Affective neuroscience generally acknowledges that basic drives arise out of dynamic topographical mapping of body states in the brain stem (Thivierge and Marcus, 2007; Damasio, 2012), which are perceived either as pleasant or unpleasant arguably ultimately depending on whether they are conducive to the maintenance of biological homeostasis (Damasio and Carvalho, 2013). This occurs through an interaction with dispositional circuits which implement these basic tendencies and thus evaluate the body states to produce valence (Panksepp, 2005). Drives provide biologically important motivational states which are purportedly re-represented in various sections of the insula, and combined with various cognitive and reward-related brain circuits in the anterior insula (Mayer, 2011). Furthermore, they influence higher cognition (such as attention) by means of the major neurotransmitter systems (i.e., dopamine, serotonin, noreadrenaline, and acetylcholine) which lead from the brain steam to various areas all over the neocortex (Bechara and Damasio, 2005). A prominent example of the influence of homeostasis on cognition is in the bidirectional communication system between the gut and the brain which makes use of interoceptive representation and has significant implications for mood, decision-making, and psychiatric disorders (Mayer, 2011).

However, drives are by far not the only determinant in the activity of the major neurotransmitter systems which are key to emotional processes, and, more generally, they are not the only factor in the multifaceted phenomena collectively referred to as emotion. It is now generally accepted that emotion is a complex phenomenon composed of motor-expressive, sensory-perceptual, autonomio-hormonal, cognitive-attentional, and affective-feeling aspects (Panksepp, 2003). Thus, it may be useful to consider an ontological category of core affect, defined as a neurophysiological state determining consciously accessible processes of pleasure and activation (Russell and Lisa, 1999). Core affect is said to be composed of various distributed circuits. Most notably, a circuit involving the basolateral complex of the amygdala, the central/lateral orbitofrontal cortex, as well as the anterior insula seems to be responsible for integrating sensory representations with higher cognition and establishing conscious perception of valenced interoceptive states. Reciprocal connections between the ventromedial prefrontal cortex, the anterior cingulate cortex, and the amygdala are thought to determine embodied emotional reactions via connections to the hypothalamus and brain stem (Barrett and Lindquist, 2008). These embodied reactions, in turn, can affect cognitive processes such as attention, memory, and learning in various ways via the major neurotransmitter systems which begin in the brainstem. Thus, for instance, norepinepherine can be utilized to focus attention and further processing on particularly relevant/salient (perhaps threatening) stimuli (Aston-Jones and Cohen, 2005). Or, as particularly relevant for Damasio's somatic marker hypothesis, dopamine signals reward prediction information to higher brain areas (Bechara and Damasio, 2005).

In order to integrate core affect into a comprehensive outline of emotion, cognitive appraisal must be taken into account. Barrett's (2014) conceptual act theory of emotion deals with this by positing that core affective states are categorized by the same domain-general systems which support other conceptualization. In this theory, top-down processing from the same ventral circuitry which establishes the core affective state directs attention toward an object which the affective state is interpreted to be about. Conversely, perception of an object triggers affective and behavioral associations by the same mechanisms, such as drawing attention to the fear-inducing stimulus and triggering the flight response. In general, cognition about the current situation based on the exteroceptive cues will modify which sensorimotor/affective states are recalled (Barsalou, 1999; Bechara and Damasio, 2005; Wilson-Mendenhall et al., 2013). On the other hand, perception and recall of affective states can be impaired. For one, affective states can take an unusual pathway and unconsciously modify behavior (Winkielman and Berridge, 2004). It can also be the case that the core affective state associated with an emotional category cannot be properly recalled, resulting in a lack of understanding of the behavioral implications of being in said emotional state—otherwise known as an empathy gap (Loewenstein, 2005). This phenomenon has been studied especially for subjects who find themselves in either a hot or a cold state, and fail to appreciate how a person would act in the opposite state. Thus, for instance, young adults make many unhealthy decisions when in a cold state because they fail to recognize how sickness would feel. In conclusion, it should be said that emotion as a whole is difficult to distinctly characterize because the division between emotion and cognition is not clear-cut, but rather gradual—many structures and processes (as mentioned above) are shared and highly interconnected (Barrett et al., 2007).

## 2. Describing emotional dynamics

The results of affective neuroscience seem to support the conclusion that emotion is an emergent phenomenon within a complex (multi-level), dynamic, and embodied system—therefore, it does not seem to lend itself to algorithmic explanation. One major blow to the view that the brain should be explained in terms of rules and representations comes from the reality that the neural structure at any of the layers of control is not fixed, but ever subject to plasticity (Mareschal et al., 2007). This means that not only are the cortical maps redrawn (and synapses modified/created, generally speaking), but also their modes of processing being adjusted subneurally in various cellular and neurochemical phenomena (Mozzachiodi and Byrne, 2009). Furthermore, (interoceptive) neural maps are commonly recognized (and highly important) organizational structures that operate dynamically in three senses: they persistently synchronize with the state of the body, are modified by plasticity, and engage in feedback loops with higher maps and cognitive areas (Petersen and Diamond, 2002; Thivierge and Marcus, 2007). Furthermore, processes relevant to emotion operate on a biochemical, systemic/neural, as well as behavioral (e.g., social) level of organization/explanation. One thus arrives at the necessity of describing (or simulating) emotional phenomena in terms of dynamical systems on multiple levels, as many parts of the brain (including cognitive ones) interact in non-linear ways and their mode of processing ever subject to feedback (Faure and Korn, 2001). Thus, emotions are in many ways an emergent phenomenon, where any given emotional episode is a gestalt arising out of the idiosyncratic and pluralistic constellation of (cognitive, embodied, and situated) processes at that given moment (Barrett et al., 2007).

## 3. The role of computation and reduction over multiple levels of measurement/organization

Progress in science unequivocally depends on a continual examination of the evidence for the existing theories. Thus, if there is to be a computational theory of the mind/brain of any scope, it must be subject to the same rigorous empirical examination that more naturalistic theories are subject to. That is not to say that there is no place for theories which are only loosely inspired by the brain, and employed to solve specific problems—as is the case in applied artificial intelligence. I intend to target, first and foremost, theories of mind/brain function and general intelligence.

**Figure 1:**
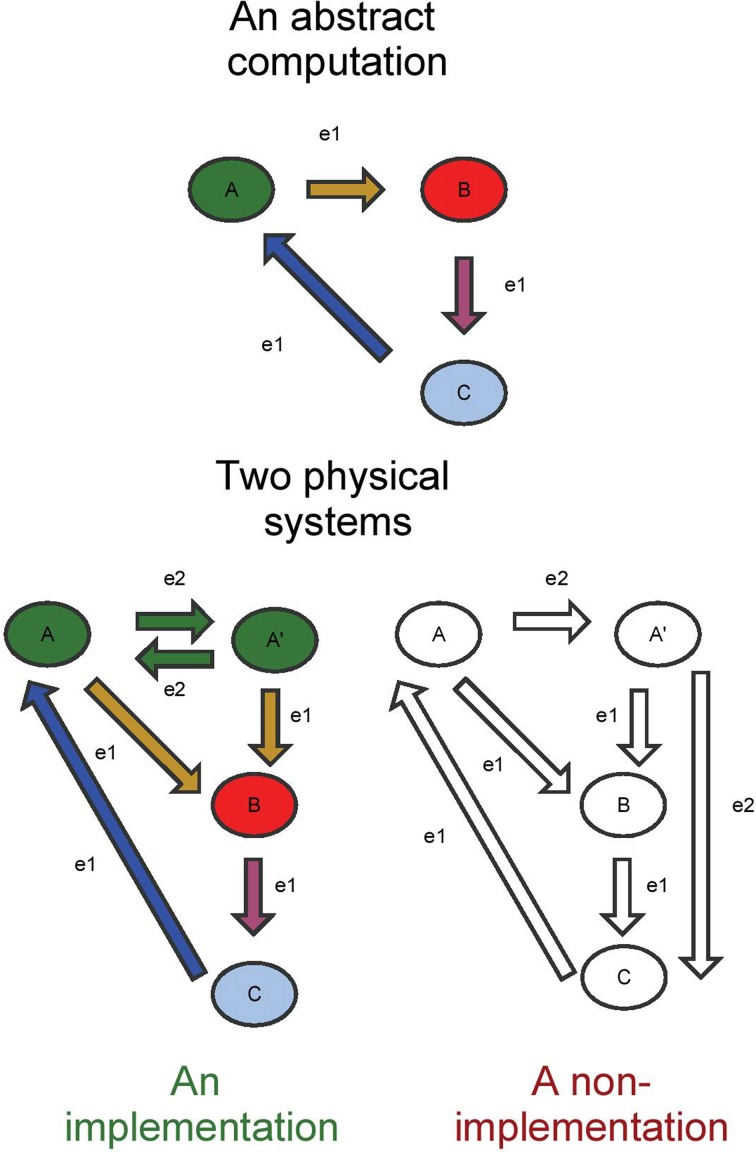
**A simple implementation and a non-implementation**. The correspondence between states/transitions is color coded for the implementation. The transitions in green in the physical system can be seen as transitions leading from the green state in the abstract system to itself. e1 and e2 indicate possible events marking the transitions between states. In the non-implementation, the fact that e2 leads away from state A′ (instead of back to A) prevents the existence of a consistent mapping to states/transitions in the abstract system.

Initially, it must be established what an acceptable computational explanation consists of. I take computational explanation to be the use of computational scientific models to explain the functional structure of a physical system, rather than merely attempting to replicate some existing physical theory in a computer. Chalmers (1996) has given a suitable (but debatable) definition of when a physical system implements an abstract computation. In essence, he states the causal structure of the physical system must reflect the causal structure of the abstract computation, including counterfactual conditions. This means that one should be able to show which (groups of) physical (brain) states correspond to which abstract (algorithmic) states, and which physical transitions correspond to which abstract transitions. The technical notion of this mapping is called an isomorphism. Figure 1 displays a very simple example of when physical states/transitions do and don't implement an abstract system, respectively.

The principles from neuroscience suggest that emotion does not admit such a description. The phenomena on the neural level (and above) are already highly complex, and the subneural (neurochemical or lower physical) phenomena additionally play a crucial role in generating emotions and attitudes. Emotions are yet another reason to suggest that the embodied brain does not operate like a traditional computer. I suggest that until implementation is shown for any aspect of the brain, computation should be seen as a tool for simulation rather than explanation. A technical elucidation of this difference can be found in Piccinini (2007) This difference is crucial, for it means that unless a functional correspondence between the brain and an abstract system can be shown, scientific theories thereof should be grounded in naturalism and its digital implementations should as closely as possible mirror the biological system. I believe this is also a lesson for (general) artificial intelligence, which could benefit from more neuromorphic architectures such as Chris Eliasmith's Spaun (Eliasmith et al., 2012).

Instead of Marr's (1982) tripartite levels of explanation explicitly serving computationalism, the mind/brain sciences should have an epistemological landscape which respects the various levels of organization at which phenomena underlying cognition exist at and emerge from de Jong (2002). In doing so, the framework of embodied cognition can be fleshed out to emphasize the common molecular/sub-molecular levels of description that the brain shares with the environment. The body is then located somewhere in between—sharing more (biological) levels with the brain than the environment, but not the highest ones necessary for cognition. In general, I contend that the study of cognition would benefit from attempting to make inter-level connections between theories. This exercise has the potential to both critically evaluate the biological viability of theories, as well as providing a mechanism for unifying previously distinct ones. If the ultimate goal of science is to be able to explain as comprehensively as possible, it is only a disservice to ignore certain levels in favor of one limited framework. Only in pluralism can one truly explain the emergent phenomenon of emotion, and more generally, cognition.

### Conflict of interest statement

The author declares that the research was conducted in the absence of any commercial or financial relationships that could be construed as a potential conflict of interest.
